# Establishing Worksite Wellness Programs for North Carolina Government Employees, 2008

**Published:** 2011-02-15

**Authors:** Suzanna Young, Jacquie Halladay, Marcus Plescia, Casey Herget, Carolyn Dunn

**Affiliations:** North Carolina Department of Health and Human Services; Department of Family Medicine, University of North Carolina, Chapel Hill, North Carolina; North Carolina Division of Public Health, Department of Health and Human Services, Raleigh, North Carolina; North Carolina State Health Plan for Teachers and State Employees, Raleigh, North Carolina; North Carolina State University, Raleigh, North Carolina

## Abstract

**Background:**

State employee health plans sometimes provide worksite wellness programs to reduce the prevalence of chronic diseases among their members, but few offer the comprehensive range of interventions recommended by the Task Force on Community Preventive Services.

**Community Context:**

North Carolina's State Health Plan for Teachers and State Employees provides health coverage for approximately 665,000 state employees, teachers, retirees, and dependents. Health claims indicate that the prevalence of having at least 1 chronic disease or of being obese is approximately 32% among state employees.

**Methods:**

The State Health Plan created a partnership with North Carolina's Division of Public Health, Office of State Personnel, and other key state agencies to identify bureaucratic obstacles to providing worksite wellness programs for state employees and to develop a state policy to address them. The Division of Public Health established a model worksite program to guide development of the worksite wellness policy and pilot wellness interventions.

**Outcome:**

The state's first worksite wellness policy created an employee wellness infrastructure in state government and addressed administrative barriers to allow effective worksite wellness interventions. For example, the policy led to pilot implementation of a subsidized worksite weight management program. Positive results of the program helped generate legislative support to expand the weight management program throughout state government.

**Interpretation:**

Strong interagency partnership is essential to guide worksite wellness policy and program development in state government. State health plans, public health agencies, and personnel agencies each play a role in that partnership.

## Background

One objective of *Healthy People 2010 *was for at least 75% of worksites to offer comprehensive worksite wellness programs for their employees (1). Worksite programs are part of a public health strategy to address the increase in chronic diseases, which are predicted to cost the US health care system an estimated $4.2 trillion annually by 2023 (2). The Task Force on Community Preventive Services recommends 18 components of an effective comprehensive worksite wellness program that fall into 4 categories: insurance benefits, policies, programs, and communications (3). Worksite programs shown to be most effective were those that used evidence-based interventions to help employees lose weight, increase physical activity, reduce tobacco use, and have better access to influenza vaccination (3).

State employee health plans are beginning to play a role in efforts to reduce the prevalence of chronic disease and contain health care costs by providing wellness programs to their members. Most state health plan wellness efforts have focused on disease management services, and few include the multiple intervention strategies recommended for effective wellness programs. State and local governments are among the largest employers and health coverage purchasers in many states (4). All 50 states offer their employees some level of health insurance coverage (5). In response to rising health care costs, some states have introduced wellness and prevention initiatives for state employees; most of these initiatives comprise only 1 or 2 strategies, such as health monitoring and assessments, insurance incentives, healthy work environment initiatives and recognition programs, and fitness challenges or events (4).

North Carolina's State Health Plan for Teachers and State Employees was one of the first state health plans to integrate worksite wellness and policy development into its health promotion and chronic disease management strategies. In 2004, faced with projected escalating costs for an increasing proportion of members with at least 1 chronic health condition, the State Health Plan expanded its role to offer prevention programs, education, and resources to members. A healthy living initiative (www.shpnc.org/nc-healthsmart.html) was created to offer prevention and care management services to members. The State Health Plan also collaborated with legislators and state leaders to improve wellness benefits and ensure more comprehensive coverage of preventive services.

The State Health Plan invited state agencies, schools, and public colleges and universities to join a partnership to promote evidence-based worksite wellness programs for state employees and identify policies and legislation needed to reduce bureaucratic barriers to those programs (6). The absence of any state policy or legislation to enable or define employee wellness programs meant that complex state regulations, policies, and procedures applied when organizing wellness interventions at state worksites. For example, farmers' markets and commercial wellness vendors that offered weight loss and fitness programs purchased directly by employees were not allowed on state property without first undergoing a long and complicated bid and contract process for competitive vendors.

This article describes how the State Health Plan 1) partnered with the Division of Public Health, Office of State Personnel (OSP), and other key state agencies to identify barriers and develop a state worksite wellness policy to support employee wellness programs, 2) created a replicable model worksite wellness program, and 3) supported a subsidized and incentivized worksite weight management project to demonstrate the effect of the state's new worksite wellness policy.

## Community Context

North Carolina's state government workforce is 66% white, 29% black, and 5% other races/ethnicities (7). The mean age of employees enrolled in the State Health Plan is 44.8 years and most (68%) employee members are women (8). North Carolina self-funds the State Health Plan rather than contracting for coverage with an insurance company; the plan provides coverage for approximately 665,000 state employees, teachers, retirees, and their dependents. Of this number, approximately 133,000 are state agency and university employees. Consistent with national trends (2), the proportion of plan members with at least 1 chronic disease increased rapidly over the past decade (9). Data from 2008 employee-only health claims show a prevalence of 32% for 1 or more of 6 chronic diseases (diabetes, coronary artery disease, chronic obstructive pulmonary disease, asthma, hypertension, and congestive heart failure). The prevalence of hypertension was 28% and for diabetes was 9% (8).

In 2009, a State Health Plan analyst categorized 62% of adult plan members as overweight or obese. Of the 32% of adult members who were obese (body mass index [BMI] ≥30 kg/m^2^), 18% had a BMI of more than 40 kg/m^2^. Obesity-related costs for plan members have been calculated at $108 million per year or $700 to $1,000 for each obese member (E. A. Finkelstein, PhD, written communication, March 4, 2009).

The Department of Health and Human Services (DHHS) is North Carolina's second largest state agency; approximately 18,000 employees work in agency office settings, mental hospitals, treatment centers, and residential schools for disabled students. In 2007, 39% of DHHS employees had a high school education or less, and 39% earned $30,000 or less per year. The DHHS employed larger proportions of women (73%) and minorities (43%) than state government overall (7). Low education and income levels and a higher proportion of minorities likely contributed to the higher prevalence of chronic diseases (39%) among DHHS employees compared with the 32% prevalence for all state employees. Department employees also had higher hospitalization rates of 60.9 per 1,000 in 2008 compared with 52.7 per 1,000 for all State Health Plan members (8). The  prevalence of overweight and obesity in 2008 was higher for DHHS employees (73%) than for all adult plan members (J. Halladay, December 1, 2009).

The goal of the state agency partnership was to reduce employee risk of developing chronic health conditions and to contain rising health care costs by establishing worksite wellness programs throughout state government. The first step was approval of a state worksite wellness policy that outlined a wellness infrastructure for state government and removed organizational barriers to effective worksite wellness interventions.

## Methods

In 2004, the State Health Plan convened a worksite wellness advisory committee, with broad representation from state government, to promote evidence-based worksite wellness programs and identify policies and legislation needed to reduce bureaucratic barriers to worksite programs for state employees. By 2005, the committee identified major barriers to wellness interventions for state employees and recommended policies and legislation to promote employee wellness and support worksite programs. These included support for incentives to promote participation in wellness activities, work time for wellness committee work, smoke-free worksites, and improved preventive screening benefits.

In 2004, the State Health Plan also created a 5-year partnership with the North Carolina Division of Public Health that 1) established a replicable model worksite wellness program under the direction of a full-time wellness director and 2) developed a worksite wellness tool kit (www.eatsmartmovemorenc.com/NCHealthSmartTlkt/WorksiteTlkt.html) as a resource for state government wellness programs, and 3) trained state entities to use the tool kit to create a wellness program for their employees.

The State Health Plan selected the DHHS for the model worksite wellness program because of its large size, diverse workforce, high prevalence of chronic diseases, and strong leadership support for an employee wellness program. The DHHS wellness program focused on changes to organizational policies and the work environment to increase support and opportunities in the workplace for physical activity, healthful eating, tobacco use cessation, and stress management. Wellness committees in 38 divisions, offices, and facilities developed and implemented wellness plans addressing the program focus areas, and a department-wide wellness council helped guide policy issues. Online surveys and focus groups identified program barriers and guided planning and wellness policy recommendations. Annual employee surveys assessed participation levels, satisfaction, and health behavior changes. Wellness committees reported on wellness activities, environmental support, and policy and administrative barriers they encountered in implementing wellness programs.

On the basis of these data, the DHHS wellness director, with support of the DHHS wellness council, recommended several policy and administrative changes to the department secretary, who immediately authorized employee access to existing fitness areas at DHHS residential facilities and 4 hours per month of work time for wellness committee work and approved fundraising activities to support wellness programs. Barriers beyond DHHS authority included a ban on employee incentives, prohibitions on using agency administrative funds for employee wellness programs, restrictions on use of space in state buildings for fitness areas, and regulations related to wellness vendors on state property.

In 2006, the State Health Plan invited executive leadership from several key state agencies to participate in creating a wellness oversight committee to develop model wellness policies specific to state government worksites. The new group replaced the large worksite wellness advisory committee and included representation from the OSP, the university system and community college administrations, the Department of Public Instruction, and later the Division of Public Health. The committee combined recommendations from the earlier advisory committee and from the DHHS wellness director to develop policy recommendations that would 1) give state agencies and universities the authority to create and support worksite wellness programs, 2) provide an infrastructure for creating sustainable wellness programs, 3) address the major state bureaucratic barriers to employee wellness programs, and 4) ensure that employees receive a uniform level of wellness services and supervisory support. Specific policy recommendations addressing each area were developed by a workgroup and formally submitted by the wellness oversight committee to OSP in 2007 as proposed additions to state personnel policy. In 2008, the North Carolina State Personnel Commission, following an agency review and public comment process, approved as submitted the recommendations as the OSP worksite wellness policy (www.osp.state.nc.us/manuals/manual99/Worksite%20Wellness%20Policy.pdf).

## Outcome

Approval of North Carolina's first worksite wellness policy increased the authority of the state and its agencies and universities to implement evidence-based interventions to prevent and control chronic disease among state government employees. The policy specifically addresses the major chronic disease risk factors by allowing changes in the workplace to increase levels of physical activity, improve access to more healthful foods, support tobacco use cessation, and reduce and manage stress. The policy also outlines a wellness infrastructure for state government that can be maintained without additional staff and calls for wellness programs to be monitored and evaluated. The strategy used by the State Health Plan to develop the worksite wellness policy has since been adopted by OSP to institutionalize the process. A full-time wellness coordinator position was established in OSP to provide state oversight of implementation of the policy and future wellness policy development. Permanent state committees, one with agency and university wellness leaders and a second with representation from a broader coalition of state, research, and private organizations, will be created to guide this process.

As a result of the new policy, a subsidized worksite weight management program was established for DHHS employees that offered a cash rebate and pedometers as incentives to participate and complete 15 weekly classes. Of the 138 employees who completed the pilot, the mean weight loss was 6.5 pounds (Table); 80% lost an average of 8.2 pounds. The proportion of participants with normal blood pressure increased from 23% to 36%. A follow-up survey found that 81% of the 111 participants who lost weight during the program reported they had maintained (48%) or lost additional weight (33%) at 6 months.

Two provisions of the policy — approval to use administrative funds (if available) to subsidize wellness activities and approval of small incentives to promote employee participation in wellness activities — contributed to the success of the intervention. Results from the pilot helped the State Health Plan gain support from the North Carolina General Assembly in 2009 for a comprehensive wellness initiative that supported expansion of the pilot to similar weight management programs across state agencies and universities.

## Interpretation

Before North Carolina created a worksite wellness policy, state agencies and universities faced several obstacles to offering worksite wellness interventions. The state's new worksite wellness policy essentially exempts wellness programs for state government employees from some state regulations and procedures that had limited the use of evidence-based wellness interventions. For example, the policy allows agencies to offer small incentives to promote employee participation and encourages state office space to be designated for wellness activities, including exercise.

State Health Plan leadership adapted its strategy during a 4-year period to drive development of the worksite wellness policy. The first wellness policy advisory committee, with broad representation from throughout state government, promoted awareness of the benefits of worksite wellness programs for state employees and identified barriers to providing evidence-based wellness interventions in state government. The committee also helped build consensus in state government on worksite wellness issues, which proved instrumental in gaining agency and university approval of the policy in the OSP policy review process. Later replacing the large advisory committee with a smaller wellness oversight committee with executive leadership from key agencies proved effective in quickly finalizing the policy recommendations, submitting them for state approval, and providing oversight for dissemination of the approved policy. Members of the wellness oversight committee considered the overall policy development strategy effective but recognized that representation on the committee from the Department of Administration and the Office of State Budget and Management could have provided added clarity and guidance on sections of the wellness policy development related to the use of state property for wellness activities and fiscal support for wellness programs.

The DHHS wellness program was a useful model for a wellness infrastructure for state government that, except for the full-time director position, could be implemented and sustained with existing staff. The OSP worksite wellness policy approval of 4 hours of work time monthly for agency wellness committee members and 6 hours for the committee chair supported wellness program development in each state agency. A designated wellness director, however, is necessary to develop and sustain a comprehensive worksite wellness program in a large state agency or university. The DHHS wellness director provided leadership and oversight to develop, implement, and evaluate the department's wellness program. The director was also able to partner with the State Health Plan to pilot wellness initiatives for potential implementation throughout state government.

**Table:** Box. Key Strategies for Worksite Wellness Policy Development in State Government

Establish a strong partnership with leadership from the employee health plan, key state agencies (eg, public health and personnel), and universities to provide oversight of worksite wellness policy development for state employees.
Recruit broad representation from state agencies and universities to serve on a worksite wellness advisory committee and focus committee efforts on 1) identifying barriers to implementation of evidence-based wellness programs in state government or employee participation in wellness activities, and 2) addressing those barriers by developing wellness policies.
Use existing state agency wellness programs to help identify state policies, administrative procedures, or regulations that impede offering effective worksite wellness interventions. Surveys or focus groups of wellness committees and employees can help identify barriers and needed policy changes. States without worksite wellness programs could benefit by implementing a worksite program in 1 large agency to help identify obstacles and to serve as a model wellness program for other state agencies.
Restructure worksite wellness advisory committees as needed to ensure they include key leadership to develop and implement wellness policy throughout state government.
Have state employee health plans, public health agencies, and personnel agencies contribute to the partnership. Establishing strong interagency partnerships similar to those we describe should not depend on which agency provides primary oversight of employee wellness programs. The choice of agency to take the lead role in organizing a partnership to drive wellness policy development appears to be unimportant.

The collaborative approach that state agencies used to develop a policy to support worksite wellness programs for North Carolina state employees provides a replicable model for most states. Organizational obstacles to wellness programs may vary among states, but the strategies recommended are applicable to most states, including those offering employee health benefit packages though multiple vendors (Box). A state's health plan entity that coordinates employee coverage under a single umbrella, not third-party health benefits plan administrators, should take the lead role with other state agencies to drive state employee wellness policy.

## Figures and Tables

**Table.** Changes in Mean Anthropometric Measures Among State Employees Participating in a Worksite Weight Management Pilot Program, North Carolina, 2008

**Measure**	n^a^	Baseline (SD)	Post-Program (SD)^b^	Difference	*P* Value^c^
Weight, lbs	138	197.4 (42.8)	190.9 (41.8)	6.5	<.001
Waist circumference, in	130	39.6 (5.3)	38.0 (5.1)	1.6	<.001
Body mass index, kg/m^2^	138	33.0 (7.0)	31.9 (6.9)	1.1	<.001
Systolic blood pressure, mm Hg	127	124.5 (14.9)	120.6 (13.5)	3.9	<.001
Diastolic blood pressure, mm Hg	127	83.8 (9.2)	80.6 (8.2)	3.2	<.001

Abbreviation: SD, standard deviation.

a Number of participants for whom baseline and post-program measures were available.

b Measured at the end of the 15-week program.

c Calculated by using *t* tests.
